# Dual‐Scale Spiral Material for Balancing High Load Bearing and Sound Absorption

**DOI:** 10.1002/advs.202400250

**Published:** 2024-03-30

**Authors:** Chenlei Yu, Mingyu Duan, Fei Ti, Fengxian Xin, Guiping Zhao, Tian Jian Lu, Runpei Yu, Moxiao Li, Xin Chen

**Affiliations:** ^1^ State Key Laboratory for Strength and Vibration of Mechanical Structures Xi'an Jiaotong University Xi'an 710049 P. R. China; ^2^ National Key Laboratory for Mechanics and Control of Aerospace Structures Nanjing University of Aeronautics and Astronautics Nanjing 210016 P. R. China; ^3^ Department of Advanced Manufacturing and Robotics Peking University Beijing 100871 P. R. China; ^4^ MIIT Key Laboratory of Multi‐functional Lightweight Materials and Structures Nanjing University of Aeronautics and Astronautics Nanjing 210016 P. R. China; ^5^ MOE Key Laboratory for Multi‐functional Materials and Structures Xi'an Jiaotong University Xi'an 710049 P. R. China; ^6^ Department of Mechanical Engineering Seoul National University Seoul 08826 South Korea; ^7^ Xi'an Modern Chemistry Research Institute Xi'an 710065 P. R. China

**Keywords:** double porosity, load‐bearing properties, multi‐functional integrated design, sound absorption

## Abstract

Porous materials with sound absorption and load‐bearing capabilities are in demand in engineering fields like aviation and rail transportation. However, achieving both properties simultaneously is challenging due to the trade‐off between interconnected pores for sound absorption and mechanical strength. Inspired by quilling art, a novel design using spiral material formed by rolling planar materials into helical structures is proposed. Experimental results show high structural strength through self‐locking mechanisms, while double porosities from interlayer spiral slits and aligned submillimeter pores provide excellent sound absorption. These spiral sheets surpass foam aluminum in specific strength (up to 5.1 MPa) and approach aerogels in sound absorption (average coefficient of 0.93 within 0–6400 Hz). With its adaptability to various planar materials, this spiral design allows for hybrid combinations of different materials for multi‐functionality, paving the way for designing advanced, lightweight porous materials for broad applications.

## Graphical abstract

A dual‐scale spiral material is introduced to strike a balance between mechanical performance and sound absorption. The dual‐scale spiral materials exhibit good mechanical performance due to the confinement effect and interlayer self‐locking mechanisms across multiple layers of planar material. Additionally, the spiral slits and large specific surface area inherent to dual‐scale spiral material offer possibilities for achieving sound absorption. Abundant raw material sources and environmental sustainability make them promising for diverse applications in households, industries, and transportation.

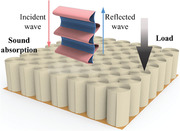

## 1 Introduction

Porous materials are widely used as sound absorbers due to their good sound absorption performance^[^ ^1^, ^2^, ^3^ ^]^ attributed to their mass transfer characteristics and large specific surface area.^[^ ^4^ ^]^ Previous efforts have primarily focused on achieving broadband and lower‐frequency sound absorption through the design of internal channel structures.^[^ ^2^, ^3^, ^5^, ^6^ ^]^ The need for simultaneously enhancing both acoustic and mechanical properties of porous materials has been less explored, which becomes increasingly critical in applications such as highway soundproof walls, concert hall floors, submarines, and aviation aircraft.^[^ ^7^, ^8^ ^]^ The challenge arises from the inherent trade‐off between the acoustic and mechanical properties, as the former demands interconnected pores while the latter thrives on a robust, solid framework. Achieving these dual functionalities in one porous material could reduce weight, thereby contributing to decreased energy consumption and progress toward carbon neutrality.

From a structural perspective, porous materials can be classified as disordered or ordered. Disordered porous materials comprise foam materials,^[^ ^8^ ^]^ granular materials,^[^ ^9^, ^10^ ^]^ and fiber‐based materials.^[^ ^3^, ^11^ ^]^ Such disordered porous material features tortuous channels that effectively enhance the friction and dissipation of sound waves^[^ ^12^ ^]^ and are typically low‐cost with facile fabrication (e.g., melt foaming,^[^ ^13^ ^]^ electrospinning powder,^[^ ^14^ ^]^ pressing,^[^ ^9^ ^]^ and sol–gel method^[^ ^2^ ^]^). However, a significant drawback is their susceptibility to local deformation caused by defects, weakening the structural strength under loading.^[^ ^15^ ^]^ In contrast, ordered porous materials (e.g., micro‐lattice) with regular microstructure and reduced defects possess enhanced mechanical properties, such as higher strength, stiffness, and energy absorption capacity.^[^ ^15^, ^16^ ^]^ With the surge of advanced manufacturing technologies (e.g., 3D printing), high‐strength porous materials have been developed by hybridizing and optimizing various lattice structures.^[^ ^17^, ^18^ ^]^ When considering the sound absorption capability, it is generally imperative to ensure that the pore size within the lattice structure is maintained at sub‐millimeter dimensions.^[^ ^19^ ^]^ However, when the pore size is reduced to such a level, the defects from interlayer bonding^[^ ^20^ ^]^ and processing inaccuracies (ranging from 0.01 to 0.3 mm),^[^ ^21^ ^]^ which are comparable in scale to the pores, become significantly important. Defects can compromise the structural strength of lattice structures,^[^ ^18^ ^]^ while errors can significantly impact the accuracy of acoustic performance prediction and design.^[^ ^7^ ^]^ Consequently, fabricating porous materials with controllable submillimeter pores for high load‐bearing and sound absorption remains challenging,^[^ ^15^ ^]^ calling for a facile and scalable design solution.

**Figure 1:**
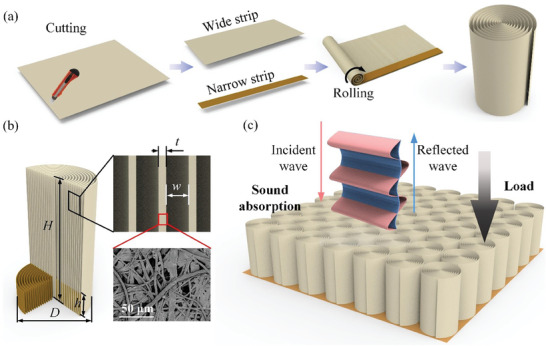
Dual‐scale spiral material for sound absorption and load‐bearing requirements a) The preparation process of dual‐scale spiral material. First, the planar material is cut into two strips of different widths, and then the two strips are affixed together and rolled up into a dual‐scale spiral material. b) Cross‐sectional view of the dual‐scale spiral material. c) Schematic diagram of the dual‐scale spiral material array for load‐bearing and sound absorption.

Inspired by the art of quilling, we propose a new design concept to fabricate ordered porous materials by spiral planar materials via cut‐and‐roll processing (**Figure** **1**). The designed material contains a spiral skeleton with uniform, controllable submillimeter pores. We rolled ordinary papers to construct spiral materials with evenly‐spaced, thin‐walled spiral structures, and realize superior structural strength and sound absorption performance comparable to closed‐cell aluminum foam and aerogel, respectively. Combining theoretical analysis, experiment, and finite element method, we elucidate the confinement effect and interlayer self‐locking mechanisms for load‐bearing, as well as the sound absorption mechanism of double porosity of the dual‐scale spiral material. Based on the spiral design concept and mechanism, we further extend this design to hybrid spiral materials made of ceramic fiber felt and aluminum foil, exhibiting high‐temperature resistance and multi‐functional integration. Finally, we compare the spiral material with other porous materials (i.e., aerogel, porous ceramic material, foamed aluminum, and natural fiber composite), and illustrate the superiorities and broad potential applications of the design concept.

## 2 Results

### 2.1 Preparation and Characterization

We fabricate the dual‐scale spiral material with ordered spiral slits by the cut‐and‐roll method shown in Figure 1a. The preparation steps are as follows: i) Cutting the planar material into uniformly wide and narrow strips; ii) Affixing a narrow strip to one end of the wide strip to govern the formation of ordered spiral slits. A wider strip's width determines the final height (*H*) of the spiral material, while a narrower strip's thickness determines the width of the slit (*w*); iii) Spiral the planar material along its length direction, transforming it into a spiral cylinder; iv) Taping the outermost edge of the wide strip to prevent unraveling.

This study uses a common planar material, e.g., paper, to prepare the spiral material. As the paper has natural pores, we obtained ordered and disordered pores at two different scales: the ordered spiral slits with *w* = 10^2^ µm, while the disordered pores of the paper with an average pore size ≈10 µm (Figure 1b). Based on this design concept, geometric parameters, such as the diameter (*D*) and height (*H*) of the cylinder, slit width (*w*), thickness (*t*), and porosity (ϕ_p_) of planar material and the height of the narrow strip, can be flexibly adjusted to fabricate various dual‐scale spiral materials (Part S2, Supporting Information). The ordered spiral paper wall provides load‐bearing capabilities along the axial direction, while the multi‐scale porosity maximizes the utilization of internal pores and enables functionalities, e.g., sound absorption (Figure 1c). Subsequent sections will discuss the influence of these design parameters on the mechanical properties and sound absorption performance.

### 2.2 Mechanical Properties

**Figure 2:**
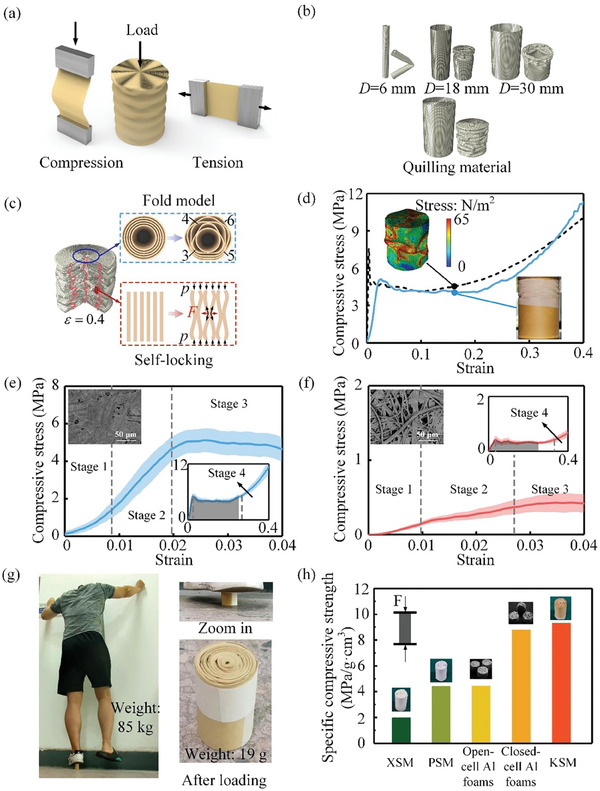
Mechanical properties of dual‐scale spiral material. a) Deformation response of single planar material through in‐plane compression and tension, with the spiral material axially loaded. b) Simulations of deformation modes of thin‐walled circular tubes with different diameters and the spiral material. c) Schematics of the axial and radial deformation constraints within the spiral material during compression. d) Comparison between the simulated stress–strain curve and experimental data of the spiral material (kraft paper) under compression. e,f) Compressive stress as functions of strain of the KSM and XSM. We conducted five sets of experiments. The solid lines represent the mean value, while the translucent shading represents the standard deviation of the five datasets. g) Photographs of the KSM supporting 4000 times its own weight, where the spiral material remains intact after loading. h) Comparison between the specific compressive strength of the spiral materials (XSM, PSM, and KSM) and aluminum foams (open‐cell and closed‐cell aluminum foams).

It is generally accepted that planar materials exhibit limited in‐plane compressive resistance due to their low bending stiffness *D* = (*Et* ^3^)/(12(1 − ν^2^)), (where *E* and ν are respectively the Young's modulus and Poisson's ratio of the planar materials), caused by the small thickness *t*.^[^ ^22^ ^]^ In contrast, planar materials demonstrate remarkable in‐plane tensile stiffness and strength (e.g., Kraft paper has a tensile stiffness of 2.12 GPa and a tensile strength of 36.1 MPa^[^ ^23^ ^]^). When subjected to in‐plane compression, a single planar material sheet is prone to buckling. However, when the planar material is rolled up and subjected to axial compression, radial expansion of the spiral material level contributed by the localized buckling of each layer, can be effectively confined by the sealed outermost edge of the planar material (**Figure** **2**a). The finite element method has been used to investigate the deformation mechanism of the spiral material under axial compression (Part S6, Supporting Information). The buckling mode depends on the diameter‐to‐thickness ratio (*D*/*t*) and the height‐to‐diameter ratio (*H*/*D*). Figure 2b presents typical buckling behaviors of thin‐walled monolithic cylindrical shells subjected to axial compression. For a given shell thickness (0.1 mm) and height (50 mm), global instability in Euler buckling mode occurs when the *D*/*t* ratio is relatively small (e.g., 6 mm/0.1 mm). As the diameter *D* increases (18 and 30 mm), the buckling behavior transitions into an asymmetric mode, accompanied by increased circumferential fold numbers.^[^ ^24^ ^]^ When an axial compressive force is applied to the spiral material, the single‐layer, thin‐walled cylindrical shell gradually buckles, eventually reaching contact with the adjacent layers. As a result, the dual‐scale spiral material becomes stabilized and cannot undergo further axial folding due to the balanced state between interlayer constraints and outermost tape confinement. We name this phenomenon as self‐locking characteristics. After the spiral material reaches this self‐locking state, additional out‐of‐plane compression is subsequently converted into in‐plane tension (the red arrow *F* in Figure 2c). This conversion significantly enhances the load‐bearing capacity of spiral material due to the good tensile strength of planar material. Figure 2d compares the experimental and numerical results of the stress–strain curves and crushing morphologies of the spiral material under axial compression. Both experimental and simulation results indicate that the spiral material exhibits typical compressive characteristics similar to porous materials. The compression plateau phase closely aligns with the simulated values, albeit slightly lower in the experimental data due to the potential non‐uniformity of slits during manufacturing. In the elastic phase, the simulation predicts higher modulus and yield stress than experimental results, attributed to the anisotropic and porous nature of paper material resulting in lower bending stiffness and strength than a uniform elastic‐plastic model would suggest. However, beyond a strain of 0.35, experimental stress values exceed simulated values due to the neglect of in‐plane stresses with the utilization of shell elements in the simulation, underestimating in‐plane contributions during densification.

The effects of the planar materials’ microstructures on the mechanical properties are investigated by using Kraft paper and Xuan paper (Figure 2e,f). We fabricated dual‐scale spiral materials with *D* = 30 mm, *w* = 0.1 mm, and *H* = 50 mm according to the preparation method in 2.1 using Kraft paper and Xuan paper, respectively. To minimize the impact of peripheral enclosing material on the mechanical properties of the spiral material, we have employed paper adhesive tape (with a thickness of 0.05 millimeters) that is similar to the paper material to secure the outer layer of the spiral material. The compression tests via universal testing machine showed that both the kraft‐paper‐derived spiral material (KSM) and the Xuan‐paper‐derived spiral material (XSM) undergo four stages (Methods, `Test’ section): the reinforcement stage, the linear elastic stage, the plateau stage, and the densification stage. In the reinforcement stage, the paper gradually deforms until adjacent layers are brought into contact, while the compressive modulus of the spiral material continuously increases. In the linear elastic stage, the deformation of the paper is constrained due to the interlayer self‐locking mechanisms. In the plateau stage, the self‐locking structure is disrupted, and the spiral material undergoes significant plastic collapse. In this process, the stress fluctuates with the increased strain around a stable value, which is attributed to the continuous formation of folds in the spiral material during the compression failure process. Following the densification stage, the stress rises steeply, and KSM behaves like solid materials due to the complete closure of spiral slits. The scanning electron microscope (SEM) images revealed that Kraft paper has more dense pores, while Xuan paper has pores with larger pore sizes. The differences in microstructures between the two papers result in higher plastic hinge strength of Kraft paper than that of Xuan paper. The compressive modulus and strength of the XSM are measured as 11 and 0.45 MPa, respectively. In contrast, KSM has a compressive modulus of 350 MPa and a compressive strength of 5.1 MPa. Considering the low weight of the material, KSM exhibits a high specific compressive strength, enabling it to withstand loads exceeding 4000 times its own weight (85 kg/0.019 kg) without significant deformation (Figure 2g). The specific compressive strength is a key parameter that quantifies the load‐bearing capacity of a material relative to its weight. By selecting materials with higher specific compressive strength, designers can optimize the structural efficiency and reduce the overall weight of the system, leading to energy savings and environmental benefits. We compared the specific strength of three types of spiral materials (KSM, XSM, and printing paper‐derived spiral material (PSM)) with commonly used open‐cell and closed‐cell aluminum foam,^[^ ^25^ ^]^ as illustrated in Figure 2h. The specific compressive strength of KSM was found to be the highest among the tested materials, surpassing that of closed‐cell foam aluminum. This superior strength is attributed to its microstructure, which is characterized by predominantly closed micrometer‐scale pores (porosity ≈0.52). Conversely, the higher porosity (0.65) of printing paper compared to Kraft paper, resulted in a lower specific compressive strength for PSM, although it remained comparable to open‐cell foam aluminum. In contrast, XSM exhibits the poorest performance due to its excessively high porosity (0.88), drastically reducing its self‐locking ability.

It is also worth noting that we observe the progressive folding mode of the spiral material in the plateau stage (up to 30% strain) (Part S5, Supporting Information). Therefore, the spiral material can act as an energy absorber when subjected to impact loads, with a specific energy absorption density of $E\left(d\right)=\int_{0}^{d}P(\varepsilon )d\varepsilon$ (ε is the compressive strain), as the shaded area shown in Figure 2d.

### 2.3 Sound Absorption Performance

**Figure 3:**
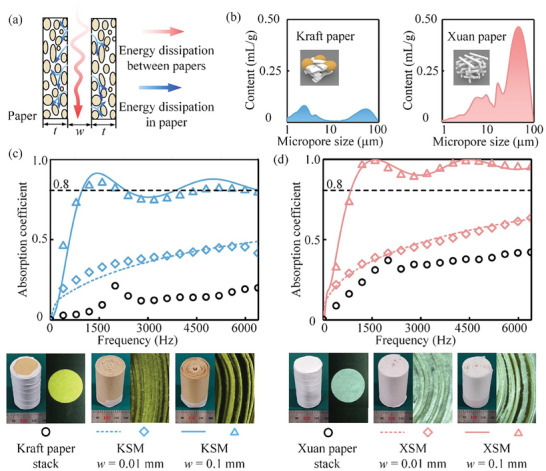
Sound absorption performance of dual‐scale spiral material. a) Schematic of the sound absorption mechanism. The sound waves propagate in the artificial slits between the paper layers and partially enter the paper through the natural micropores in the paper. b) Pore size distribution of Kraft paper and Xuan paper. We conducted measurements on four mixed samples obtained from different locations on two separate sheets of paper. c) Sound absorption coefficient as functions of frequency for Kraft paper stack and KSMs with *w* = 0.01 mm and *w* = 0.1 mm. d) Sound absorption coefficient as functions of frequency for Xuan paper stack and XSMs with *w* = 0.01 mm and *w* = 0.1 mm. The experimental data are the average of multiple measurements from five specimens. Scatters and curves in (c) and (d) are experimental data and theoretical predictions, respectively.

The dual‐scale spiral materials have two interconnected networks with different shapes and characteristic sizes (i.e., dual‐pore structure): spiral slits (approximately hundreds of micrometers) and natural micro‐pores in paper (approximately tens of micrometers). The internal pores of paper are smaller than the thickness of the viscous boundary layer within the measured range (<6400 Hz), resulting in significant flow resistance and hindered penetration of sound waves.^[^ ^6^ ^]^ Consequently, the sound absorption performance of the paper is negatively affected. However, the dual‐pore structure provides an unobstructed pathway for fluid flow to enhance the mass transfer capability of the dual‐scale spiral materials, thanks to the presence of spiral slits. When a sound wave arrives on the upper surface of the spiral material, it pushes air into the spiral slits. The friction between the vibrating air molecules and the slit walls converts partial sound energy into thermal energy (red arrow in **Figure** **3**a). Subsequently, the sound wave traverses spiral narrow slits and infiltrates the micro‐pores of the paper, inducing air vibrations in the micro‐pores, further increasing the dissipation of energy (blue arrow in Figure 3a).

This sound absorption effect can be predicted by the homogenization theory of the double‐porosity model.^[^ ^5^, ^6^ ^]^ First, the paper is assumed to be completely non‐porous at the first scale, and a flow model is established for the spiral slits. Then, at the second scale, the flow model within the micropores of the paper is computed. Finally, the coupled flux between two scales is derived to obtain an equivalent homogenization model for the daul‐scale spiral material. However, the dual‐pore structure provides an unobstructed pathway for fluid flow to enhance the mass transfer capability of the dual‐scale spiral materials. The relatively small contrast in size between the micropores within the paper and the spiral slits leads to a pronounced inter‐pore coupling effect. Due to the thinness of the paper, the sound pressure is evenly distributed along the direction of wave propagation. The average pressure within the micropores of the paper is nearly equal to that in the neighboring regions of the spiral slits. Thus, the dual‐pore structure provides an unobstructed pathway for fluid flow to enhance the mass transfer capability of the dual‐scale spiral materials can be characterized using mixing rules.^[^ ^26^ ^]^

(1) $$\rho_{\mathrm{eff}}={\left(\phi_{s}\frac{1}{\rho_{s}}+\left(1-\phi_{s}\right)\frac{1}{\rho_{p}}\right)}^{-1}$$

(2) $$K_{\mathrm{eff}}={\left(\phi_{s}\frac{1}{K_{s}}+\left(1-\phi_{s}\right)\frac{1}{K_{p}}\right)}^{-1}$$

where ϕ_s_ = *l*_s_/(*l*_s_ + *l*_p_) is the volume fraction of narrow slits in the whole spiral material. ρ_s_ and *K*_s_ are the density and compression coefficient of the slit with non‐porous wall. ρ_p_ and *K*_p_ are the density and compression coefficient of porous material using Johnson–Champoux–Allard (JCA) equivalent fluid mode. For detailed information, please refer to the “Sound absorption module” outlined in the Methods section.

The macroscopic characteristic impedance *Z*_eff_ and wavenumber *k*_eff_ of the However, the dual‐pore structure provides an unobstructed pathway for fluid flow to enhance the mass transfer capability of the dual‐scale spiral materials can be deduced as:

(3) $$Z_{\mathrm{eff}}=\sqrt{K_{\mathrm{eff}}\rho_{\mathrm{eff}}}$$

(4) $$k_{\mathrm{eff}}=\omega \sqrt{\rho_{\mathrm{eff}}/K_{\mathrm{eff}}}$$

In the presence of a rigid acoustic backing, the surface acoustic impedance rate of the dual‐scale spiral materials can be expressed as:

(5) $$z_{s}=Z_{\mathrm{eff}}\cdot \coth\left(jk_{\mathrm{eff}}h\right)/Z_{0}$$

where *Z*_0_ = ρ_0_ *c*_0_ is the characteristic impedance of air, ρ_0_ is the density and *c*_0_ is the sound speed in air.

Correspondingly, its sound absorption coefficient α is:

(6) $$\alpha =1-{\left|\frac{z_{s}-1}{z_{s}+1}\right|}^{2}$$

We conducted Mercury Intrusion Porosimetry analysis (Experimental Section, ’Material Characterization’) to characterize the pore structure of Kraft paper and Xuan paper. Kraft paper, which is reinforced with coarse fibers and fillers, exhibits a low porosity characterized by a bimodal distribution of pore sizes ≈3 and 50 µm (Figure 3b). In contrast, Xuan paper utilizes thinner fibers to fulfill the demand for permeability in writing applications, so it has a higher porosity with a pore size concentrated ≈50 µm (the porosity with a pore size of 50 µm is nine times higher than that of Kraft paper). Although there are natural micropores inside a paper, both Kraft paper and Xuan paper cannot achieve satisfactory sound absorption only by themselves. We stacked paper sheets with a diameter of 30 mm into cylinders with a thickness of 50 mm to test the sound absorption effect of the paper itself. The experimental results indicated that the average sound absorption coefficient of the Kraft paper stack is measured to be 0.12, with a maximum value of 0.22 (black circles in Figure 3c). Xuan paper stack with larger porosity exhibits an average sound absorption coefficient of 0.32 and a maximum sound absorption coefficient of 0.4 (black circles in Figure 3d). The sound absorption peaks of paper stacks at ≈1900 Hz can be attributed to the resonance caused by the gaps between the stacked paper sheets.

Then, we tested the sound absorption coefficients of KSM and XSM with different slit sizes *w* = 0.01 mm (diamond symbols) and *w* = 0.1 mm (triangle symbols), as shown in Figure 3c,d. It can be observed that the predictions obtained through the double‐porosity theory (curves in Figure 3c,d)) align well with our experimental data (material parameters are in Part S1, Supporting Information). The average sound absorption coefficient from 0 to 6400 Hz of KSM without any added narrow paper slips (*w* = 0.01 mm) reaches 0.3. Notably, the value of *w* = 0.01 mm corresponds to an inherent manufacturing error that unavoidably arises during the fabrication process. This value was ascertained through inverse analysis based on the measured sound absorption performance of the spiral material. We further enlarged the slit size to *w* = 0.1 mm by adding a narrow paper slit made of printing paper. Then, the average sound absorption coefficient of KSM with *w* = 0.1 mm increases to 0.8 mm. However, achieving perfect sound absorption (*α* > 99%) remains challenging for KSM since only the viscous dissipation through spiral slits is limited. Fortunately, XSM serves as an ideal solution to the issue of perfect sound absorption. Compared with KSM, the average sound absorption coefficient of XSM increases from 0.3 to 0.45 with *w* = 0.01 mm and from 0.8 to 0.93 with *w* = 0.1 mm. It is worth noticing that the sound absorption coefficient of KSM with *w* = 0.1 mm is beyond 0.8 for all frequencies higher than 850 Hz and achieves a maximum coefficient of 0.99 at 1420 Hz. The above remarkable sound absorption enhancement of XSM can be attributed to the high porosity of Xuan paper, which is measured to be 0.88. In addition, the sound absorption performance of the dual‐scale spiral materials can be regulated by tailoring the spiral slit width *w* and the overall thickness *h* (Part S2, Supporting Information).

### 2.4 Dual‐Scale Spiral Material with Periodically Perforated Paper

**Figure 4:**
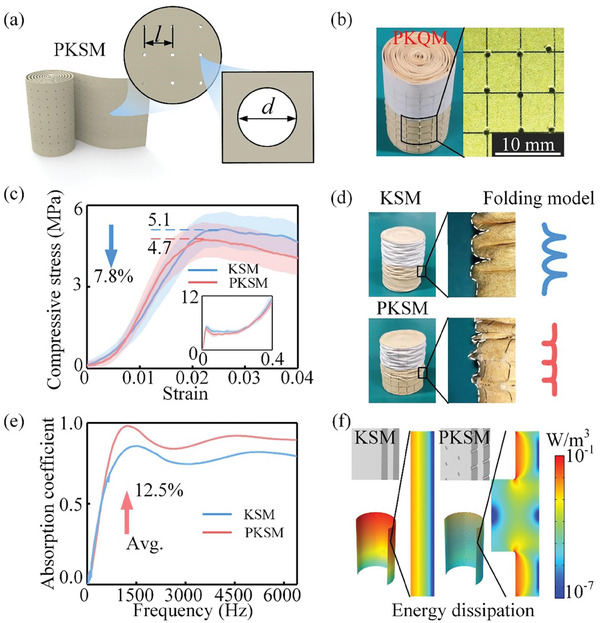
Mechanical properties and sound absorption performance of dual‐scale spiral material made of Kraft paper with artificially ordered pores. a) Schematic of spiral material with artificially ordered pores. *l* represents the spacing between pores, and *d* represents the diameter of the pore. b) Photograph of perforated Kraft paper‐derived spiral material (PKSM), where the pores have a diameter of *d* = 0.6 mm and are perforated by a syringe needle with a grid size of *l* = 5 mm. c) Compressive stress as functions of strain for PKSM and KSM. Five sets of experiments were conducted. The solid lines denote the mean values, whereas the translucent shading indicates the standard deviation. d) Deformation modes of PKSM and KSM under axial compression. The red and blue curves represent the deformation of the outermost layers of PKSM and KSM, respectively. e) Sound absorption coefficient as a function of frequency for PKSM and KSM. f) Energy dissipation density distributions of PKSM and KSM at peak absorption frequency simulated by commercial finite element software COMSOL Multiphysics.

To combine the advantages of XSM with better sound absorption performance and KSM with good mechanical strength, we further proposed a new design strategy to create ordered micropores in the dual‐scale spiral material through artificial periodic perforation on non‐porous planar materials (**Figure** **4**a). The ordered distribution of artificial pores reduces the effect of disordered pores on the structural stiffness and strength of porous materials, as well as provides additional pores for the realization of improved sound absorption. Taking the Kraft paper as an example, we first drew a 5 mm × 5 mm grid on the paper and then orderly perforated the grid nodes using a 0.6 mm‐diameter syringe (Figure 4b). Subsequently, we fabricated the perforated Kraft paper‐derived spiral materials (PKSM) with *w* = 0.1 mm through the process illustrated in Figure 1.

Compression tests show that the reduction in structure stiffness caused by the perforation process is negligible (Figure 4c), as the self‐locking mechanism eliminates the effect of ordered pores on the overall structural deformation. The local load‐bearing capacity near the ordered micropores in the Kraft paper is reduced after the perforation, which causes a decrease in structural strength from 5.1 MPa (KSM) to 4.7 MPa (PKSM), with a reduction of 7.8%. A transition in the buckling mode is observed under axial compression from asymmetric (in KSM) to axisymmetric (in PKSM) due to the initial crushing near the perforated cross‐section area (Figure 4d). The blue and red curves in Figure 4d depict the contour lines of KSM and PKSM crushing specimens, respectively. In the PKSM specimen, artificially ordered pores at the same horizontal position form a plastic hinge line and generate circumferential strain. The folding formation and energy absorption are influenced by the pores' size and density, allowing us to modulate the dual‐scale spiral material's energy absorption by adjusting these parameters.

Acoustic tests showed that incorporating artificially ordered pores into Kraft paper significantly improved the sound absorption performance of the dual‐scale spiral material for a frequency band beyond 500 Hz (Figure 4e). Compared to KSM, the peak frequency of the sound absorption coefficient for PKSM decreased from 1560 to 1184 Hz, with PKSM achieving a quasi‐perfect sound absorption peak (*α* = 0.982). Moreover, the average sound absorption coefficient of PKSM below 6400 Hz is improved by 12.5%. Numerical simulations of the energy dissipation density in PKSM and KSM reveal that the perforations in Kraft paper introduce additional solid‐gas interfaces that contribute to sound energy dissipation (Figure 4f). The artificial pores further increase the air molecular vibration velocities of airflow in spiral slits, enhancing the energy dissipation density in spiral slits. In conclusion, artificially introducing ordered pores in the dual‐scale spiral material significantly improves sound absorption performance, while maintaining a good load‐bearing capacity.

### 2.5 Hybrid Spiral Material

**Figure 5:**
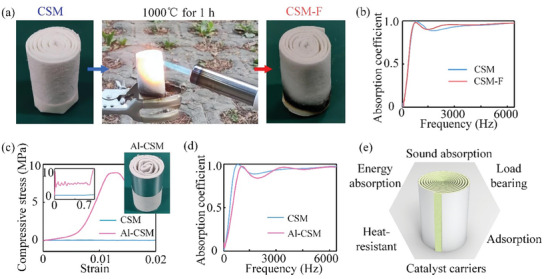
Hybrid spiral material. a) Photographs of ceramic fiber felt‐derived spiral material (CSM) before and after the heat treatment for 1 h by a butane blowlamp. The thickness of ceramic fiber felt is *t* = 1 mm, and the slit width is *w* = 1 mm. b) Sound absorption coefficient as a function of frequency for CSM and heat‐treated CSM (CSM‐F). c) Compressive stress as functions of strain for CSM and Al‐CSM, in which Al‐CSM features an aluminum foil with a thickness of 0.15 mm, one single experiment was conducted. d) Sound absorption coefficient as functions of frequency for hybrid spiral materials Al‐CSM and CSM. e) Schematic for hybrid spiral materials with multi‐functionalities.

To demonstrate the broad applicability of the spiral design concept, we further incorporated planar materials with superior intrinsic functionalities (such as ceramic fiber felts) to design spiral materials. Ceramic fiber felts are a multi‐functional material known for their abundant internal porosity and ceramic properties that enable attractive characteristics, including fire resistance, heat resistance, filtration capability, and sound absorption.^[^ ^2^, ^27^ ^]^ By employing the aforementioned design concept, we developed ceramic fiber‐derived spiral materials (CSM) with *h* = 50 mm, *D* = 30 mm, and *w* = 1 mm, as shown in **Figure** **5**a. The high porosity of ceramic fiber felts contributes to the sound absorption performance of CSM, with a sound absorption coefficient beyond 0.8 for the frequency band above 500 Hz and an average sound absorption coefficient of 0.91 for 0–6400 Hz (blue curve in Figure 5b). To verify the fire and heat resistance of CSM, we used a butane torch to heat‐treat CSM at a flame temperature above 1000 °C for one hour. Both CSM and heat‐treated CSM show sound absorption performance superior to that of XSM and KSM. The sound absorption performance of CSM after heat treatment is ≈100% retained (red curve in Figure 5b).

However, CSM displays negligible load‐bearing capacity in the compression test (blue curve in Figure 5c), which is due to the brittleness of ceramic fibers and the numerous disordered pores inside the felts, resulting in a low plastic hinge strength formed in CSM and consequently weakening the load‐bearing capacity of the material. To address this issue, we introduced a 0.15 mm‐thick aluminum foil as a reinforcement and spiraled ceramic fiber felt and aluminum foil together to construct a hybrid spiral material (Al‐CSM). The Al‐CSM exhibits better mechanical properties than that of XSM and an excellent sound absorption performance. The compression test for Al‐CSM shows a significant increase in modulus (1640 MPa) and strength (8.2 MPa). Additionally, the retention rate of sound absorption performance of Al‐CSM exceeded 95% after introducing aluminum foil (green curve in Figure 5d). The hybrid spiral design concept will also provide new insights into the porous material design for broader application fields, such as adsorption, heat transfer, and catalysis (Figure 5e). It is noteworthy that the performance of aluminum will be affected by the high temperature, and the mechanical properties of Al‐CSM will inevitably decrease after high temperature. Possible approaches include considering alternative metals with higher melting points (e.g., titanium and iron) and implementing surface coating treatments for enhanced heat resistance.^[^ ^28^ ^]^

## 3 Discussion

**Figure 6:**
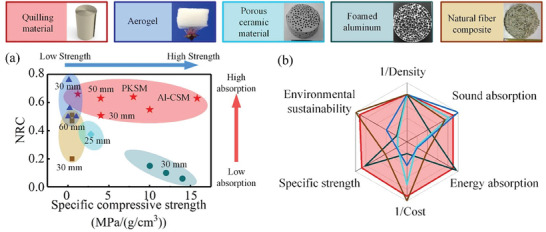
Comparative analysis of comprehensive performance between dual‐scale spiral material and other porous materials. a) An Ashby diagram showing the specific compressive strength versus NRC of the spiral material with other porous materials of differing thicknesses: Aerogel. Reproduced with permission.^[^ ^2^ ^]^ Copyright 2020, Springer Nature. Porous ceramic material, Reproduced with permission.^[^ ^30^ ^]^ Copyright 2012, Elsevier. Foamed aluminum. Reproduced with permission.^[^ ^32^ ^]^ Copyright 2000, Acoustical Society of America. Nature fiber composite. Reproduced with permission.^[^ ^33^ ^]^ Copyright 2015, Elsevier. b) A radar chart comparing the features of spiral material to those of the other main classes of porous materials.

We conducted a comparative study on the sound absorption properties and mechanical performances of the dual‐scale spiral material and other commonly used porous materials (i.e., aerogel, porous ceramic material, foamed aluminum, and natural fiber composite) (**Figure** **6**a).^[^ ^2^, ^25^, ^29^, ^30^, ^31^, ^32^ ^]^ The acoustic performance of porous materials can be evaluated using the Noise Reduction Coefficient (NRC), which represents the average sound absorption coefficient at 250, 500, 1000, and 2000 Hz. The mechanical performances are assessed using specific compressive strength. The *x*‐axis represents the specific strength of the materials, while the *y*‐axis represents the sound absorption performance. Aluminum foam has the highest specific strength (5–15 MPa (g cm^−3^)^−1^)^[^ ^31^ ^]^ but relatively poor sound absorption performance (NRC is ≈0–0.3),^[^ ^32^ ^]^ whereas aerogel has the best sound absorption performance but the weakest mechanical strength.^[^ ^2^ ^]^ For homogenous materials, heightened thickness traditionally corresponds to heightened sound absorption efficacy.^[^ ^32^ ^]^ In a bid to fortify the scholarly rigor of our investigation, we have introduced a fresh ensemble of performance metrics pertaining to materials boasting a 30 mm thickness. Our analysis reveals that, under equivalent thickness parameters, the proposed spiral material maintains a competitive edge in balancing sound absorption and load‐bearing attributes. This distinctive advantage can be attributed to its submicron‐level spiral porous structure, originating from the spiral narrow slit design.

The overall evaluation of dual‐scale spiral material is further illustrated in a radar chart to further compare factors used to select material in advanced product design in practical application (Figure 6b; details shown in Part S10, Supporting Information). Six characteristics are selected to evaluate the materials based on performance requirements, sustainability, and cost‐effectiveness: density, sound absorption performance, energy absorption capacity, cost, specific strength, and environmental friendliness. With density held constant, it is found that spiral material can effectively balance high‐performances across all six criteria. Dual‐scale spiral materials exhibit excellent performance in terms of sound absorption, with an NRC of 0.7 comparable to most porous sound‐absorbing materials. Thanks to its unique spiral design, the dual‐scale spiral material demonstrates outstanding load‐bearing and energy absorption capabilities. However, we must acknowledge that foamed aluminum has superior energy absorption abilities due to its higher strain capacity.^[^ ^31^ ^]^ From an economic standpoint, the spiral material has lower costs due to its wide range of raw material sources and relatively simple processing techniques, especially when compared to foam aluminum and aerogels.^[^ ^13^ ^]^ For environmental sustainability, the spiral material can be produced using recyclable or natural biodegradable materials, meeting environmental requirements. Additionally, the production process of spiral material consumes less energy and is non‐toxic, making it environmentally friendly.

It is worth mentioning that a cylindrical shape was utilized in this study as a test sample to propose and validate concepts. Other shapes can also be fabricated depending on specific environmental constraints. We compared the compressive behavior of spiral materials with four different cross‐sectional shapes (circular, triangular, square, and petal‐shaped) with the same surface area, paper thickness, and slit width. The results indicate that the spiral materials with other shapes still exhibit similar compressive characteristics. Among them, the spiral material with a circular cross‐section demonstrates the best performance during the compression process. This is attributed to the capacity of the circular configuration for uniform stress distribution and enhanced inter‐layer interaction. In contrast, the presence of sharp angles in other shapes tends to concentrate stress and cause premature failure (Part S7, Supporting Information). In our model design, we assumed uniform gaps within the porous structure, contrasting with the non‐uniform gaps observed in actual manufactured samples. To quantify this non‐uniformity, we introduced the concept of eccentricity for each layer of paper rolls and investigated its impact on the mechanical properties of spiral materials. The results indicate that eccentricity‐induced variations in gap size compromise structural integrity near wider gaps, initiating wrinkling deformation during compression and thus reducing the mechanical performance of the spiral material. Moreover, we observed a direct correlation between the degree of non‐uniformity and the extent of mechanical performance degradation (Part S8, Supporting Information).

While dual‐scale spiral materials demonstrate good sound absorption and load‐bearing performance, they inherently possess higher strength in the axial direction compared to other directions. This characteristic may limit its standalone application in certain engineering scenarios. A promising approach to address this is by integrating spiral materials with honeycomb structures to form a hybrid structure. The honeycomb sandwich structure is a high‐specific flexural stiffness configuration that combines a reinforced honeycomb core and upper and lower panels. The incorporation of spiral material effectively suppresses deformation or fracture in the honeycomb walls, thereby enhancing the structural strength of the honeycomb sandwich structure. Perforation on the upper panel promotes fluid circulation, thereby utilizing the sound absorption functionality of the spiral material. Such a hybrid design can effectively enhance multi‐directional load‐bearing capacities, thus addressing a broader range of engineering demands. Compared to conventional foam materials, spiral materials offer superior load‐bearing and sound absorption capabilities at a more cost‐effective price, having the potential to replace traditional foam materials in scenarios requiring both functionalities. In architectural acoustics, spiral materials can be strategically placed to adjust reverberation time and improve speech clarity. In industrial noise control, they can be used on equipment factory walls to reduce noise propagation. In transportation engineering, they can enhance passenger comfort by being applied to car doors or engine compartments. The alignment of spiral materials' performance with the noise frequency ranges encountered in targeted scenarios, which still needs further research to transfer laboratory work into practical implementation.

## 4 Conclusion

In conclusion, we have introduced a conceptual approach to material design as an ordered porous material (i.e., dual‐scale spiral materials) at the sub‐millimeter scale, aiming to balance mechanical performance and functionality. Experiments and analysis showed that the dual‐scale spiral materials exhibit good mechanical performance due to the confinement effect and interlayer self‐locking mechanisms across multiple layers of planar material. Additionally, the spiral slits and large specific surface area inherent to dual‐scale spiral material offer possibilities for achieving sound absorption. Using ordinary paper as a raw material for testing, we demonstrated that the mechanical strength of the spiral paper surpasses that of foam aluminum with the same density yet offers sound absorption effects similar to aerogels. Furthermore, we extended the application of this study by integrating ceramic fiber paper with thermal insulation and fire‐resistant properties, as evidenced by a performance retention rate of 100% after high‐temperature burning. Moreover, the issue of load‐bearing capability in ceramic fiber materials was addressed by hybridizing them with aluminum foil. This dual‐scale spiral material, with a multitude of advantages, including abundant raw material sources, renewability, and environmental sustainability, holds potential across a wide range of applications, from household use to industrial and transportation needs.

## 5 Experimental Section

### Material Characterization

Mercury intrusion porosimetry (MIP) is a widely accepted technique for characterizing pore size distribution and fluid transport properties of porous materials.^[^ ^34^ ^]^ The principle of this technique is based on the non‐wetting nature of mercury on solid surfaces, where pressure is applied to force mercury intrusion into the pores of the porous material, overcoming capillary resistance. Consequently, the pore volume corresponding to the respective pore size can be determined by measuring the amount of mercury that enters the pores at different applied pressures. The tests were carried out using the AutoPore IV porosimeter from Micromeritics, USA.

Three types of paper, namely Kraft paper, printing paper, and packaging paper, are selected for analysis. Key parameters such as viscous characteristic length, tortuosity, density, static flow resistance, and porosity were evaluated (Figure S1d–f, Supporting Information). The viscous characteristic length is used to describe the viscous effects at intermediate to high acoustic frequencies and represents the average size of micropores at a macroscopic scale. Tortuosity refers to the deviation of the actual sound propagation path from a straight line, reflecting the complexity of internal micropores. Static flow resistance represents the resistance encountered by air molecules when passing through the material and can be expressed as the ratio of a pressure gradient to airflow velocity under steady airflow conditions. These parameters are crucial for characterizing the acoustic performance of paper, particularly in calculations based on the double‐porosity sound absorption theory.

### Sound Absorption Module

The homogenization method is based on the assumptions that 1) the sound pressure level is weak, the fibrous skeleton is treated as a completely rigid structure, and 2) the sound wavelength is much larger than a representative elementary volume (refers to the unit formed by a slit and adjacent paper sheets). Under these assumptions, according to generalized Darcy's law, the paper, spiral slits, and dual‐scale spiral material can be equivalently considered as a homogeneous fluid.^[^ ^35^ ^]^

Because the slit width of the spiral material is significantly smaller than the curvature radius, we employed a simplified 2D model for analysis. To simplify the calculations, we assumed a uniform distribution of slit and paper widths. Based on the findings reported by Stinson et al.,^[^ ^36^ ^]^ the effective density and compression coefficient of narrow slits can be determined:

(7) $$\rho_{s}=\rho_{0}{\left[1-\frac{\tanh\left(\sqrt{j}\lambda_{s}\right)}{\sqrt{j}\lambda_{s}}\right]}^{-1}$$

(8) $$K_{s}=\gamma P_{0}{\left[1+\left(\gamma -1\right)\frac{\tanh\left(\sqrt{j\mathrm{Pr}}\lambda_{s}\right)}{\sqrt{j\mathrm{Pr}}\lambda_{s}}\right]}^{-1}$$

where $\lambda_{s}=l_{s}\sqrt{\omega \rho_{0}/\eta }/2$ reflects the ratio of slits to viscous boundary layer thickness $\delta =\sqrt{2\mu_{c}/\left(\omega \cdot \rho \right)}$, and $\mathrm{Pr}=\eta C_{p}/\kappa$ is the Prandtl number.

The JCA equivalent fluid model is used for airflow inside the paper.^[^ ^37^ ^]^

(9) $$\rho_{p}=\frac{\alpha_{\infty }\rho_{0}}{\phi_{p}}\left[1+\frac{\sigma \phi_{p}}{j\omega \alpha_{\infty }\rho_{0}}\sqrt{1+j\frac{4\alpha_{\infty }^{2}\eta \rho_{0}\omega }{\sigma^{2}\Lambda^{2}\phi_{p}^{2}}}\right]$$

(10)

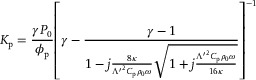

which is a semi‐phenomenological model determined by five parameters: porosity ϕ_p_, tortuosity α_∞_, static airflow resistivity σ, viscous characteristic length Λ, and thermal characteristic length Λ′. These parameters can be obtained by experimental measurements. In addition, density ρ_0_ = 1.23kg/m^3^, dynamic viscosity η = 1.85 × 10^−5^Pa s, specific heat capacity *C*_p_ = 1000J/(kg K), thermal conductivity κ = 0.026W/(m K), and the ratio of specific heat γ = 1.4 are five constants at constant pressure *P*_0_ = 1.013 × 10^5^Pa.

### Finite Element Analysis

Mechanical simulations were performed using the commercial finite element software ABAQUS. A multi‐layer, thin‐walled cylindrical shell model was employed to simulate the paper‐derived spiral material, as illustrated in Part S6 (Supporting Information). The diameter of the circular tubes increased by 0.22 mm from the innermost layer to the outermost layer, resulting in a total of 60 layers. Each layer had a thickness of 0.12 mm. Geometric nonlinearity was considered, with rigid fixtures set at the top and bottom. The bottom end was fixed, while the top end was subjected to displacement loading. A friction factor of 0.3 was considered between all layers to account for their interaction during compression. The simulations aimed to investigate the interaction between layers during the compression process.

Acoustic simulations were performed using the commercial finite element software COMSOL, utilizing the thermo‐acoustic module to model the air slit between the seams. To simplify computational complexity, we selected a single seam for simulation (Part S9, Supporting Information). Acoustic waves were incident vertically from the upper surface, with an applied sound pressure of 0.2 Pa. For structures with periodic perforations, small thermo‐viscous regions connected to the seams were used to simulate the pores.

### Tests

The quasi‐static compression test of the paper‐derived spiral material was conducted using the MTS‐880 electro‐hydraulic servo universal testing machine (Part S5, Supporting Information). The displacement load was applied at a rate of 1 mm min^−1^. The compressive modulus was determined as the slope of the stress–strain curve within the elastic region.

Acoustic experimental tests were conducted using the Brüel & Kjær 4206 impedance tube testing system, following the ASTM standard test methods, as indicated in Part S4 (Supporting Information). The distance between the two sensors was set at 50 mm. With an inner diameter of 30 mm, the impedance tube allowed for precise testing within the frequency range of 100–6400 Hz, with sampling intervals of 2 Hz.

## Conflict of Interest

The authors declare no conflict of interest.

## Supporting information

Supporting Information

## Data Availability

The data that support the findings of this study are available in the supplementary material of this article.
