# Supplementary material for: Dual‐Scale Spiral Material for Balancing High Load Bearing and Sound Absorption
Source: Adv Sci (Weinh). 2024 Mar 30;11(22):2400250. doi: 10.1002/advs.202400250 (PMC11165468; doi:10.1002/advs.202400250)
Supplement: Supplementary file 1 — Supporting Information [file ADVS-11-2400250-s001.docx]

**Supplementary materials for “****Dual-scale Spiral Material for Balancing High Load Bearing and Sound Absorption”**

**Chenlei Yu^1,2^, Mingyu Duan^3^, Fei Ti^2,4^, Fengxian Xin^1,5^, Guiping Zhao^1,5^, Tian Jian Lu^2,4^, Runpei Yu^1,2^, Moxiao Li^6^,* and Xin Chen^7^,**[[1]](#footnote-1)*

*^1^**State Key Laboratory for Strength and Vibration of Mechanical Structures,*

*Xi’an Jiaotong University, Xi’an 710049, P.R. China*

*^2^National Key Laboratory for Mechanics and Control of Aerospace Structures,*

*Nanjing University of Aeronautics and Astronautics, Nanjing 210016, P.R. China*

*^3^Department of Advanced Manufacturing and Robotics,*

*Peking University, Beijing 100871, P.R. China*

*^4^MIIT Key Laboratory of Multi-functional Lightweight Materials and Structures,
Nanjing University of Aeronautics and Astronautics, Nanjing 210016, P.R. China*

*^5^MOE Key Laboratory for Multi-functional Materials and Structures,*

*Xi’an Jiaotong University, Xi’an 710049, P.R. China*

*^6^Department of Mechanical Engineering, Seoul National University,*

*Seoul, 08826, South Korea*

*^7^Xi’an Modern Chemistry Research Institute, Xi’an, 710065, P.R. China*

**Part 1: Microstructure and acoustic parameters of papers**

Here, we selected three common types of paper: Xuan paper, Printing paper, and Kraft paper. Among them, Xuan paper has the highest porosity, followed by Printing paper, while Kraft paper has the lowest porosity. Differences in porosity among these papers are attributed to their distinct functional requirements and manufacturing processes. **Figures S1(a-c)** present the microstructures and pore size distribution of Xuan paper, Printing paper, and Kraft paper, as characterized via scanning electron microscopy (SEM). Xuan paper, commonly used for writing and painting, necessitates larger porosity and pore sizes for optimal ink penetration and water diffusion. Printing paper, extensively used for printed media, has lower porosity compared to Xuan paper due to the inclusion of fillers such as talcum powder to achieve surface smoothness. Kraft paper, a preferred choice in the packaging industry, demands robust strength and wear resistance, necessitating the tightest fiber textures, the smallest porosity, and the smallest pore size among three types of paper.

To further compare the three types of paper, key parameters such as viscous characteristic length and tortuosity thickness, density, static flow resistance, and porosity were evaluated (**Figure S1(d-f)**). Viscous characteristic length is used to describe the viscous effects at medium and high acoustical frequencies, which is an average size of the micropores at the macroscopic scale. Tortuosity is the deviation between the actual and straight lines of sound wave propagation paths, reflecting the complexity of internal micropores. Xuan paper was found to have the smallest thickness and density, followed by Printing paper, while Kraft paper was the densest and thickest (**Figure S1(e)**. Furthermore, static flow resistance and porosity were examined (**Figure S1(f)**). Air flow resistance is the resistance encountered by an air particle passing through a material, which can be expressed as the ratio of pressure gradient in the material to the airflow linear velocity under steady airflow conditions. A systematic analysis of these parameters aids in elucidating the sound absorption mechanism in involute material, thereby enhancing the scientific rigor of the research.


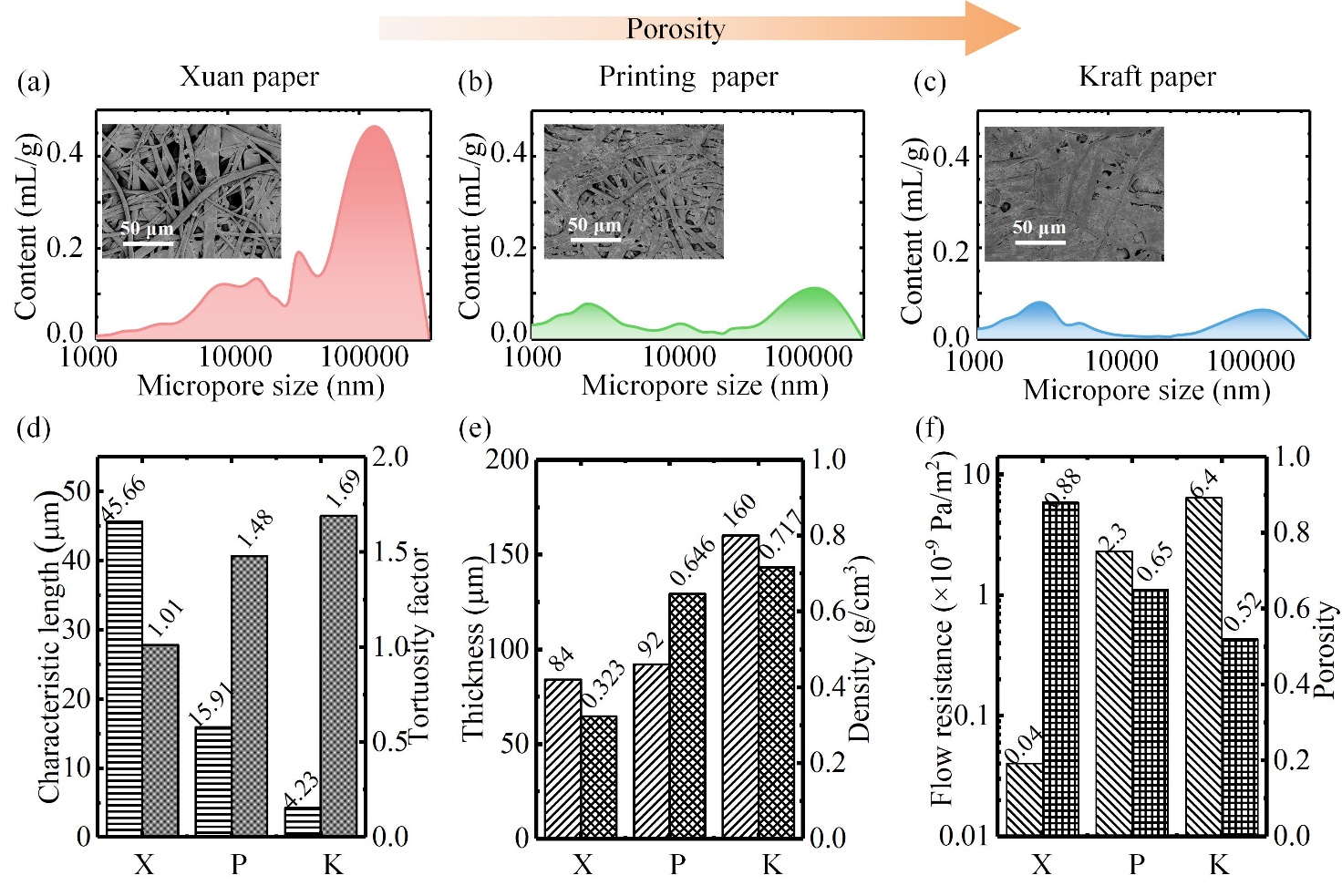


**Figure S1 Microstructure and acoustic parameters of paper and spiral material.** Microstructure and pore distribution of (a) Xuan paper, (b) Printing paper, and (c) Kraft paper. Comparison of acoustic parameters and structural parameters of the three papers. (d) Characteristic length and tortuosity factor, (e) thickness and density, and (f) flow resistance and porosity.

**Part 2: Influence of geometric structure on sound absorption coefficient of dual-scale spiral materials**

In this part, we present two sets of experiments designed to investigate the effect of various structural parameters on the sound absorption performance of spiral material made of the same type of paper. Specifically, we varied the thickness of the overall structure and the width of the slits while controlling other key factors, such as temperature and humidity, to ensure accurate results. The analysis of experimental data indicates a clear shift of the sound absorption coefficient towards lower frequencies with increasing thickness of the structure (**Figure S2(a)**). This finding can be explained by the fact that sound waves take a longer route inside the structure, thereby making the absorption of lower frequencies much more effective. Moreover, as we increased the width of the slits, we noticed a consistent decline in the overall sound absorption performance of the structure (**Figure S2(b)**). This is further compounded by the fact that the first peak shifted towards higher frequencies, a trend attributed to the lack of viscous dissipation of air against the paper wall when paper wall with wider slit.


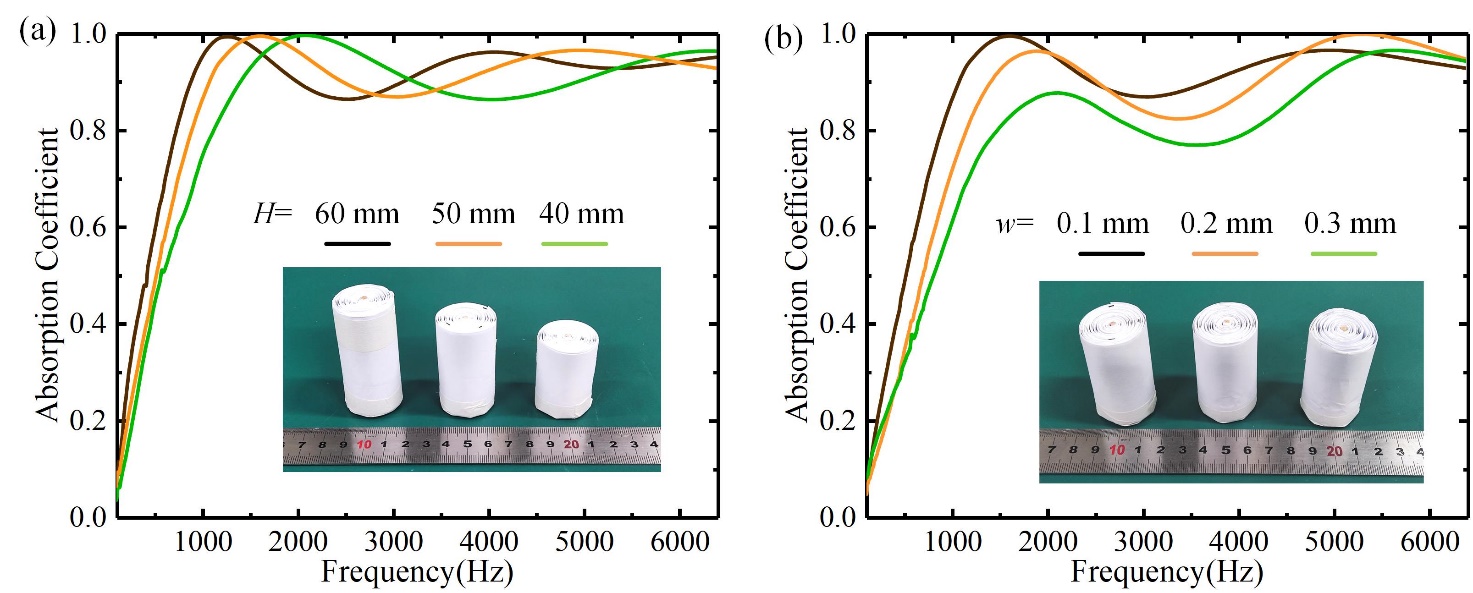


**Figure S2:** **The influence of geometric structure on the sound absorption coefficient of spiral material, using Printing paper as the selected material.** Specifically, two key factors were examined: the thickness of the overall structure and the width of the slits. (a) Three thicknesses (60 mm, 50 mm, and 40 mm) were selected with a controlled slit of 0.1 mm to study the effect of the overall structure thickness on the sound absorption performance of the spiral material. (b) Three slit widths (0.1 mm, 0.2 mm, and 0.3 mm) were selected with a controlled thickness of 50 mm to study the effect of the slit width on the sound absorption performance of the spiral material

**Part 3: Different shapes of spiral material**

The structures examined in this study were restricted to cylindrical forms, a limitation stemming from the capabilities of the available testing equipment. Nevertheless, it is essential to recognize that practical applications demand flexibility, and non-circular structural geometries may be required for specific purposes. The malleability of spiral materials presents a noteworthy advantage, as they can easily be shaped to meet varying specifications, underscoring the benefits of spiral techniques in materials engineering. To address the need for assessing shapes beyond cylindrical structures, we designed and fabricated three distinct structures, each possessing typical shapes, as exemplified in **Figure S3**. The acoustic and mechanical properties of structures with different shapes will inevitably be influenced by variations in the width of their slits. This is due to the fact that when sound waves pass through narrow slits, their frequency and amplitude will be restricted and affected, leading to an imbalanced acoustic performance and thus affecting the overall functional performance of the structure. Moreover, the mechanical properties of paper will also be affected by the curvature changes caused during deformation. This effect originates from the energy conversion during the crushing process and results in an uneven stress distribution due to the curvature changes.


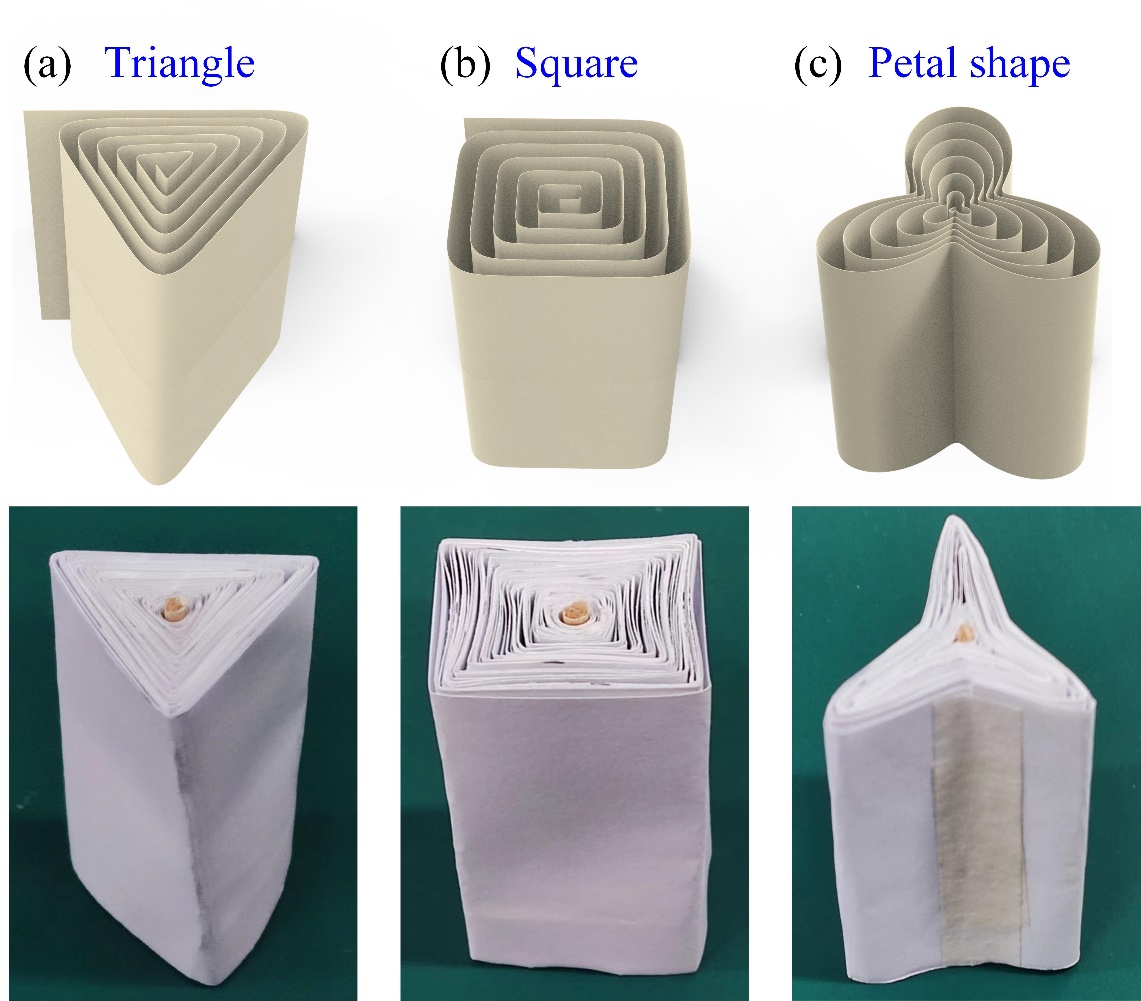


**Figure S3: Three-dimensional models of different shapes of spiral material and actual preparation of specimens**. (a) Triangle, (b) square and (c) petal shape.

**Part 4:** **Details on B&K 4206 impedance tube and PULSE Acoustic Material Testing software**

The standing wave tube method is widely used for measuring the sound absorption performance of materials. As seen in **Figure S4**, when sound waves propagate in a standing wave tube, they reflect back from the surface of the material being tested, resulting in the formation of a standing wave field. The amplitude of the sound field can be obtained by placing detection microphones at different positions within the tube and recording the maximum and minimum sound pressures at each location. Additionally, the reflection coefficient *R* of the test material can be calculated, which represents the ratio of the amplitude of the reflected wave to that of the incident wave, enabling the determination of the sound absorption coefficient *α* of the material and thus its sound absorption performance. It should be noted that the results of the standing wave tube method are only applicable to vertical incidence situations. For sound absorption characteristics under other incidence angles, alternative testing methods need to be employed. The specific measurement procedures involve fixing the sample material to be tested at the end of the standing wave tube, then radiating sound waves into the tube using a loudspeaker to propagate along the tube. Subsequently, detection microphones are placed at different positions within the tube, and the maximum and minimum sound pressures at each location are recorded using corresponding instruments. Then the transfer function between the two microphones obtained from the sound pressure signal measured by the microphone is

$H_{12}=\frac{p_{2}}{p_{1}}=\frac{e^{jkh}+R\cdot e^{-jkh}}{e^{jk\left(h+s\right)}+R\cdot e^{-jk\left(h+s\right)}}$ (S1)

where $R$ is the sound pressure reflection coefficient, $s$ and $h$ are the distance between two microphones and the distance from the second microphone to the sample surface, respectively. Thus, the sound pressure reflection coefficient is obtained by

$R=\frac{H_{12}-e^{-jks}}{e^{jks}-H_{12}}e^{j2k\left(h+s\right)}$ (S2)

Finally, the sound absorption coefficient can be obtained according to the definition:

$\alpha=1-\left|R\right|^{2}$ (S3)


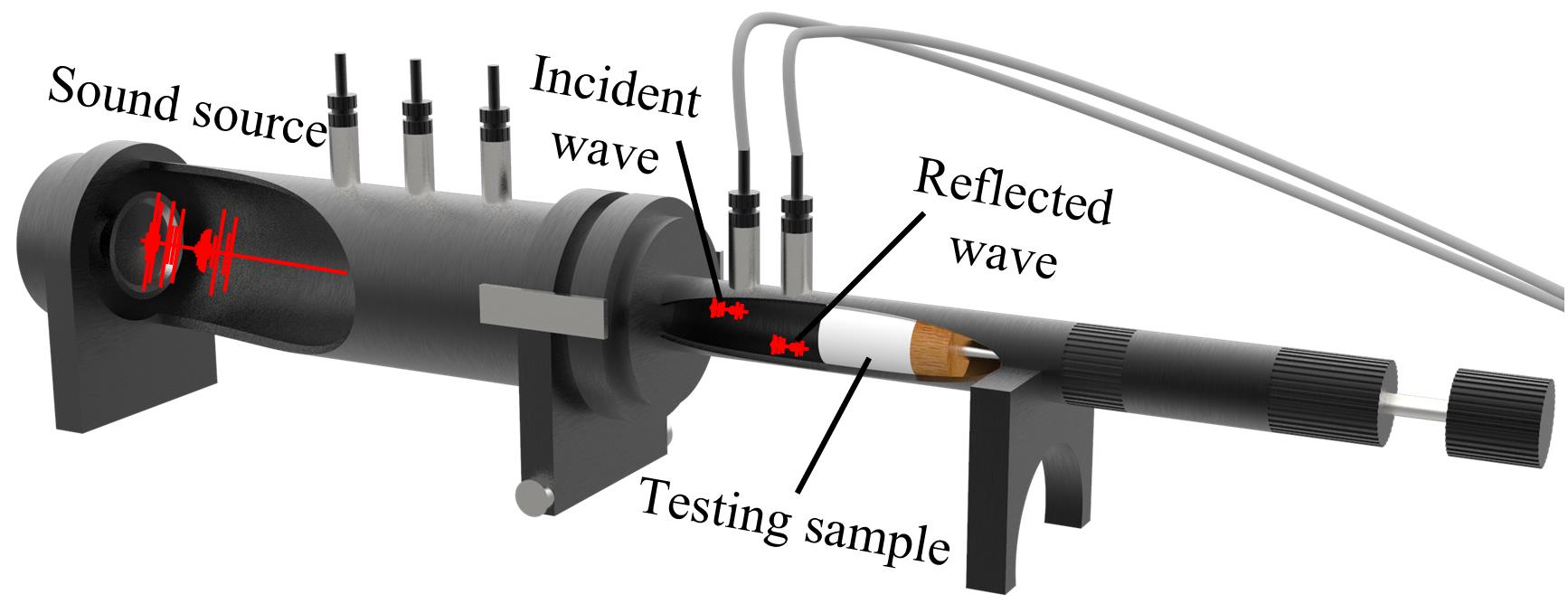


**Figure S4:** Schematic diagram of B&K 4206 impedance tube

**Part 5:** **Compression process**

The present study investigates the mechanical behavior of spiral material under quasi-static compression using a Servo-Hydraulic universal testing machine, as illustrated in **Figure S5(a)**. The testing was conducted with the lower plate fixed while the upper plate was subjected to a displacement loading rate of 1 millimeter per minute. The stress-strain curve was obtained using a mechanical sensor, as illustrated in **Figure S5(b)**. The morphologies of the compressed spiral materials are presented in **Figure S5(c)**, with an intriguing observation regarding the staggered fish scale crush on the sides of the three spiral materials. It can be observed that the surface morphology of the spiral material after compressive collapse is consistent between the portion with the adhesive tape and the portion without the adhesive tape. This observation indicates that the influence of the paper adhesive tape as a peripheral enclosing material on the deformation mode of the material during the compressive collapse process can be deemed negligible. Notably, the Kraft paper-derived spiral material displays a denser scale formation than the Printing paper or Xuan paper-derived ones, attributable to the inherent strength of the paper itself. Moreover, our analysis demonstrates that during the compression process, mesoscopic slits between the papers are initially filled, followed by the squeezing of pores between the papers, leading to increased stiffness and modulus. The crushing process of the spiral materials is analogous to that of circular tube structures. Additionally, to provide a clear representation of the compression process, we draw the compression curve using five characteristic points, as shown in **Figure S5(d)**.


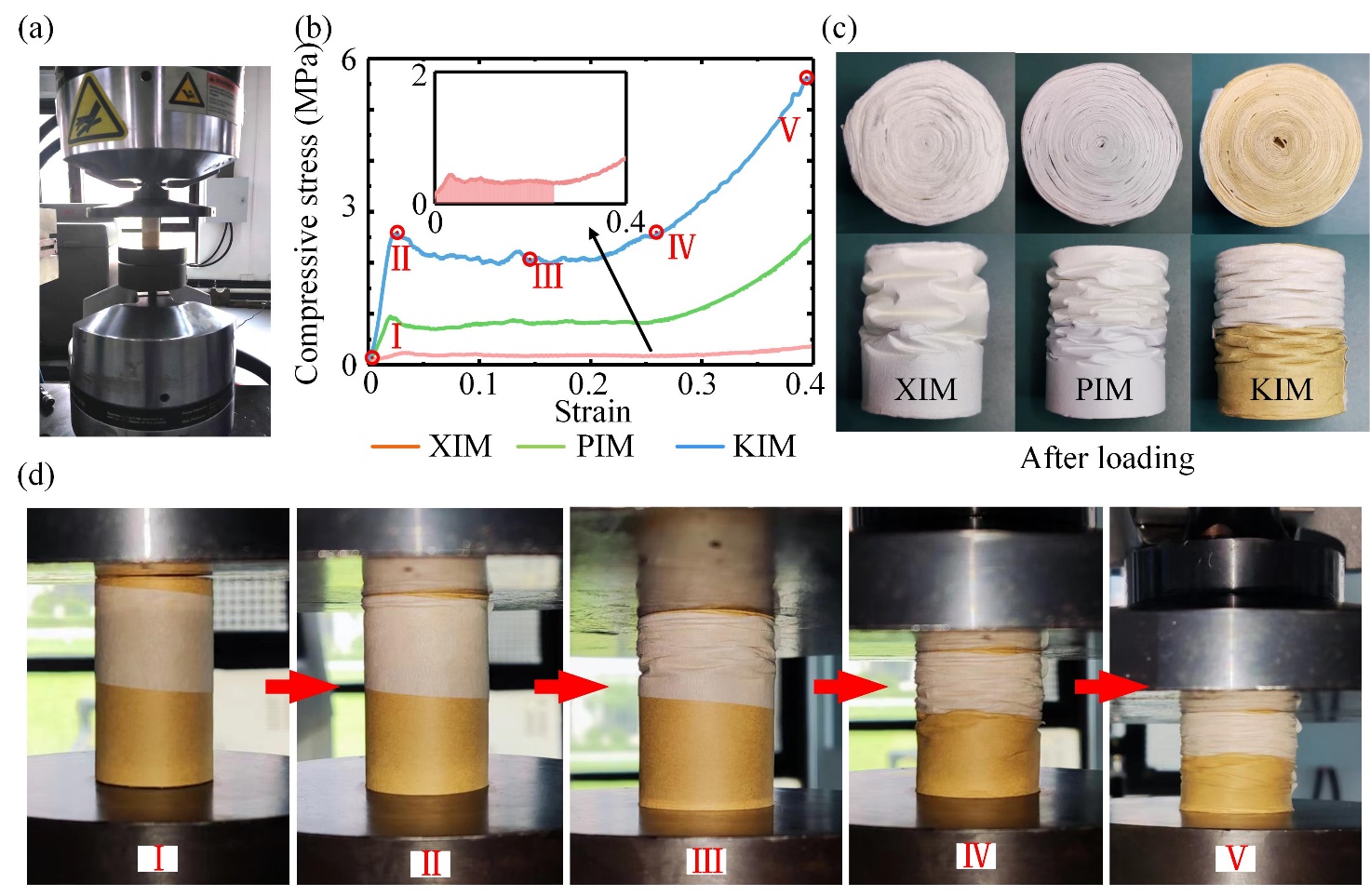


**Figure S5: Comprehensive demonstration of the quasi-static compression experiment.** (a) MTS-880 electro-hydraulic servo universal testing machine utilized for executing the quasi-static compression tests on spiral materials. (b) The compressive stress versus strain plot for the spiral materials featuring three distinct paper types. (c) Photographs of the compressed spiral materials from top and front views. (d) The five stages during the compression process correspond to five points on the stress-strain curve.

**Part 6:** **Compression finite element details**

Specifically, we analyzed the mechanical behavior of the spiral material undergoing crushing using ABAQUS/EXPLICIT software. A fully realistic simulation was impractical due to the intricate nature of the structure, leading to the use of a simplified model. The layered composition of the spiral material was represented by multiple concentric circles of paper and modeled using shell elements, with a specified gap distance of 0.22 mm. Considering the anisotropic and porous nature of paper, characterized by complex elastic and plastic deformation, we treated the material as an elastic-plastic substance, incorporating parameters such as an elastic modulus of 4 GPa, a Poisson's ratio of 0.3, a yield strength of 0.05 GPa, and a thickness of 0.12 mm based on the Mises yield criterion. Both upper and lower-end pressure plates in the model were treated as rigid bodies, with the lower plate fixing all degrees of freedom and the upper plate allowing only translational motion in the z-direction.

In the crushing analysis, a displacement load was applied to the upper-pressure plate along the negative z-axis direction, ensuring a rate slow enough to mimic the quasi-static compression process. The finite element model employed four-node reduced integration shell elements (S4R) to discretize the spiral material, with a mesh size of 1 mm × 1 mm to balance convergence and computational efficiency.

**Figure** **S6(a)** and **Figure** **S6(b)** present the longitudinal and transverse cross-sections of the compressed structure at a strain of 0.3, visually demonstrating the deformation and stress distribution during the process. The images reveal that when the inner and outer paper rolls come into contact during compression, the longitudinal direction experiences significant restraint, while the transverse section exhibits distortion. This behavior highlights the strength that multiple layers of paper rolled together exhibit compared to a single layer of paper. When considering a single layer of paper, the deformation behavior during compression varies based on the diameter of the spiral material. Specifically, for thin-walled cylindrical shells with small diameters, the deformation mode is characterized by global buckling. In contrast, while for thin-walled cylindrical shells with larger diameters, it involves local buckling of the structure, which can be seen in **Figure S6(c)**. Interestingly, multiple cylinders with different diameters lead to the constraint of internal buckling by the outer layers, while the local buckling is limited by the inner layers. This self-locking mechanism is responsible for the exceptional mechanical properties exhibited by spiral material.


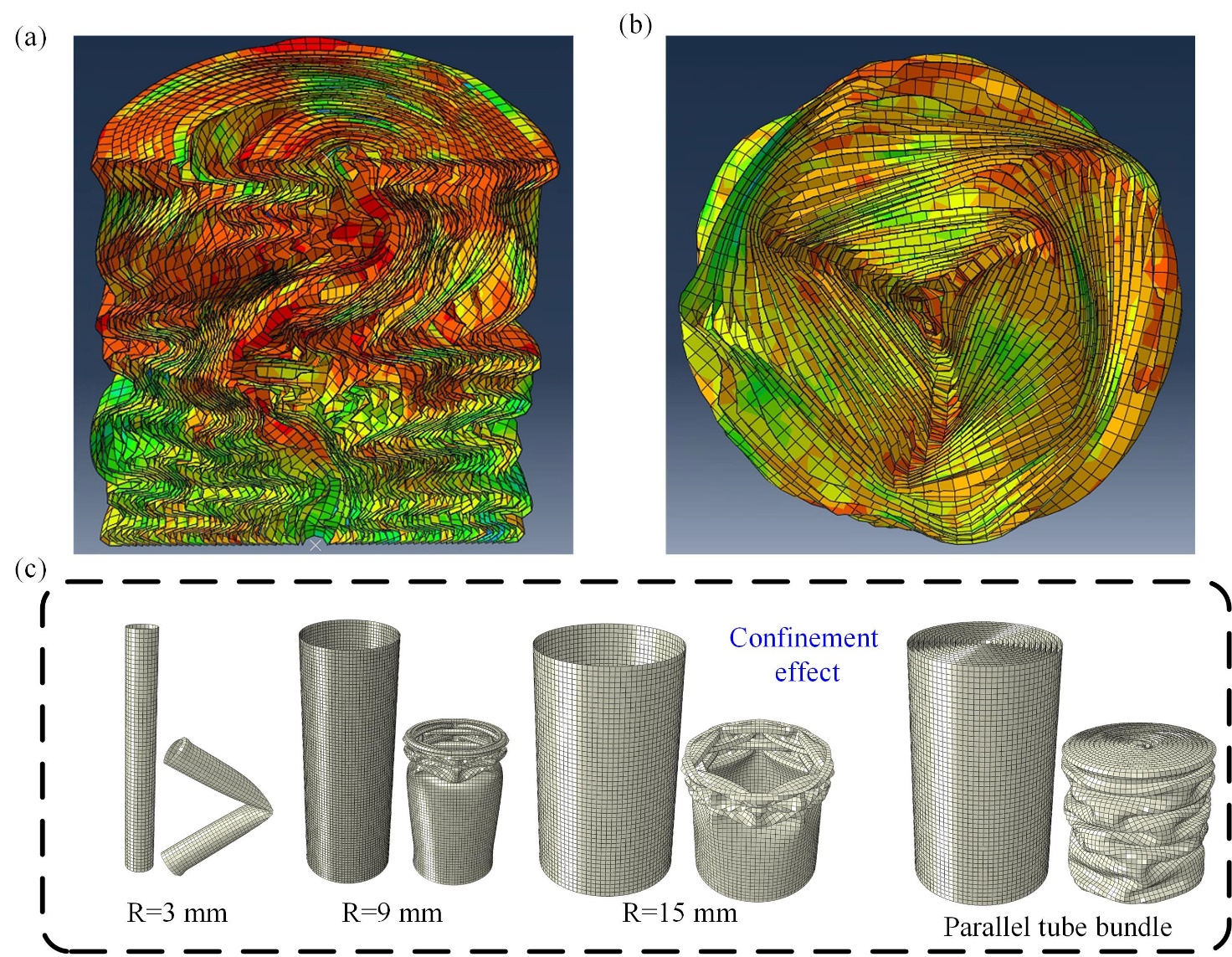


**Figure S6:** **Compression finite element details**. (a) The longitudinal and (b) transverse cross-sections of the compressed spiral materials at a strain of 0.3. (c) The compression simulation of round pipes with varying diameters and spiral materials. These simulations enable us to examine the local and global buckling behavior of the spiral materials and the self-locking mechanism imposed by the multilayer, thin-walled cylindrical shells.

**Part 7: Effect of cross-sectional shapes on the compressive performance of spiral material**

**Figure S7(a)** shows spiral materials with four different cross-sectional shapes, including circular, triangular, square, and petaled shapes. These configurations were carefully designed to have identical surface area, paper thickness, and slit width. Validated ABAQUS/Explicit models were used to analyze their mechanical properties.

The compressive stress-strain curves of the four structures are plotted in **Figure S7(b)**. The paper structure with a circular cross-section has the highest peak strength, followed by the petaled cross-section, then triangular and square cross-sections. The unsatisfying performances of triangular and square shapes are attributed to their sharp corners, which are prone to stress concentrations under uniaxial compression, leading to earlier failure. Structures with circular and petaled cross-sections, characterized by curved surfaces, benefit from more uniform stress distributions during compression, resulting in higher peak stresses.

Notably, in the densification stage after the strain exceeds 0.25, the stress of circular and petal-shaped cross-section spiral materials increases rapidly with the increase of strain, while square and triangular cross-section spiral materials have longer platform segments. To explain this phenomenon, **Figure S7(c)** compares the post-compression cross-sectional profiles of square and circular spiral materials, alongside deformed contour diagrams from identical locations. It can be observed that the square cross-section spiral material has fewer creases formed but larger deformation during compression, while the circular cross-section spiral material exhibits more creases and smaller deformation. This is because of the enhanced inter-paper coupling in circular structures, where varying diameter paper rolls mutually restrict further deformation. In contrast, the square structures, marked by weaker coupling, allow papers to move relatively freely, necessitating a smaller force for deformation as strain increases.


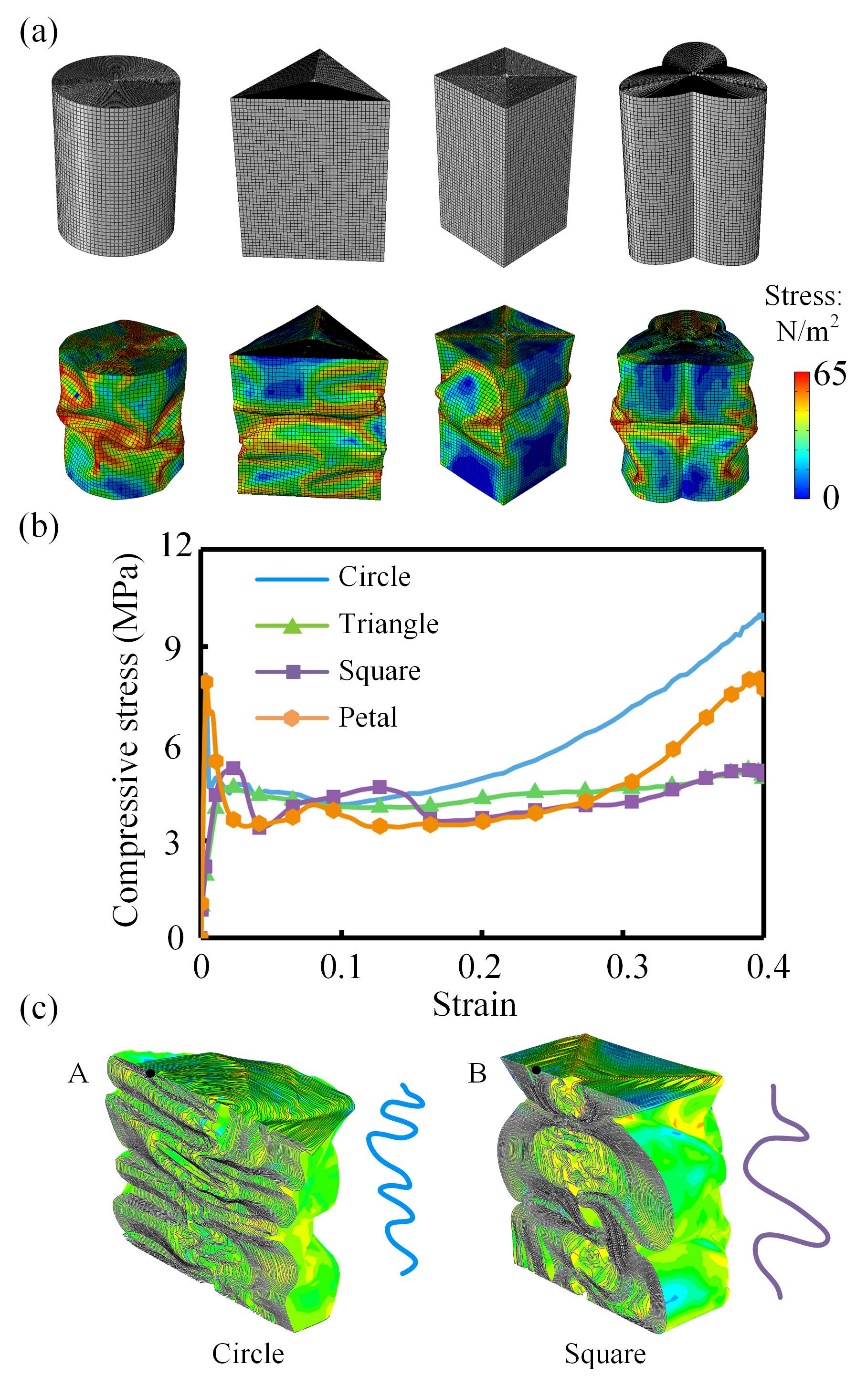


**Figure S7: Comparison of the compressive performance of spiral material with different shape configurations.** (a) Initial state and compressive deformation of spiral material with circular, triangular, square, and petaled cross-sectional shapes at a strain of 0.2. (b) The compressive stress-strain curves of the four structures. (c) Comparison of the cross-sectional profiles of square and circular spiral materials after compression

**Part 8:** **Effect of non-uniformity on the compressive performance of spiral material**

Non-uniformity is quantitatively characterized by the statistical standard deviation. To model processing-induced errors in gap spacing, we adjusted and simulated the eccentric positions of each layer of spiral material. The mechanical properties were analyzed using validated ABAQUS/Explicit models, with the details outlined as follows:

Initially, Matlab was used to generate two sets of random numbers. The first set determined the eccentricity angles of the center point for each paper layer, ranging from 0-360 degrees. The second set controls the eccentricity radius of the center for each paper layer, which is randomly generated by a Gaussian distribution to ensure an average value of 0. We studied the effect of different degrees of non-uniformity on structural performance by adjusting the standard deviation $\sigma$. A larger standard deviation indicates greater non-uniformity, while a standard deviation of 0 represents a uniform distribution. To ensure no overlap between the paper layers, the control range of the random numbers was limited to less than 0.1 mm.

Two sets of non-uniform spiral material with different standard deviations ($\sigma=0.25$ and $\sigma=0.5$) were designed to compare the effect of non-uniformity on structural performance. All specific eccentricity parameters are detailed in **Tables 1** and **2**, with the numbering sequence arranged from the innermost layer outward. **Figure S8(a)** visually displays the eccentricity distribution under different standard deviations, clearly showing a more uneven distribution for the standard deviation of 0.5 compared to 0.25.

**Table S1** Distribution of eccentric positions of paper layers based on standard deviation $\sigma=0.25$

| **No.** | 1 | 2 | 3 | 4 | 5 | 6 | 7 | 8 | 9 | 10 |
| --- | --- | --- | --- | --- | --- | --- | --- | --- | --- | --- |
| **Angle(°)** | 334 | 206 | 179 | 303 | 331 | 154 | 277 | 21 | 16 | 226 |
| **Offset Radius（mm）** | 0.032 | 0.001 | 0.019 | 0.015 | 0.022 | 0.030 | 0.034 | 0.015 | 0.039 | 0.019 |
| **No.** | 11 | 12 | 13 | 14 | 15 | 16 | 17 | 18 | 19 | 20 |
| **Angle(°)** | 5 | 16 | 48 | 243 | 247 | 89 | 2 | 295 | 226 | 97 |
| **Offset Radius（mm）** | 0.013 | 0.051 | 0.017 | 0.025 | 0.023 | 0.039 | 0.015 | 0.005 | 0.040 | 0.013 |
| **No.** | 21 | 22 | 23 | 24 | 25 | 26 | 27 | 28 | 29 | 30 |
| **Angle(°)** | 104 | 76 | 317 | 73 | 23 | 19 | 161 | 340 | 261 | 334 |
| **Offset Radius（mm）** | 0.032 | 0.049 | 0.031 | 0.002 | 0.027 | 0.018 | 0.033 | 0.021 | 0.018 | 0.038 |
| **No.** | 31 | 32 | 33 | 34 | 35 | 36 | 37 | 38 | 39 | 40 |
| **Angle(°)** | 104 | 10 | 237 | 293 | 144 | 49 | 36 | 328 | 179 | 163 |
| **Offset Radius（mm）** | 0.023 | 0.004 | 0.053 | 0.014 | 0.042 | 0.016 | 0.021 | 0023 | 0.037 | 0.004 |
| **No.** | 41 | 42 | 43 | 44 | 45 | 46 | 47 | 48 | 49 | 50 |
| **Angle(°)** | 328 | 164 | 278 | 215 | 73 | 283 | 87 | 123 | 8 | 291 |
| **Offset Radius（mm）** | 0.001 | 0.004 | 0.005 | 0.012 | 0.013 | 0.026 | 0.028 | 0.022 | 0.038 | 0.052 |
| **No.** | 51 | 52 | 53 | 54 | 55 | 56 | 57 | 58 | 59 | 60 |
| **Angle(°)** | 64 | 140 | 177 | 174 | 284 | 230 | 15 | 98 | 345 | 75 |
| **Offset Radius（mm）** | 0.003 | 0.032 | 0.004 | 0.015 | 0.020 | 0.013 | 0.025 | 0.024 | 0.009 | 0.019 |

**Table S2** Distribution of eccentric positions of paper layers based on standard deviation $\sigma=0.5$

| **No.** | 1 | 2 | 3 | 4 | 5 | 6 | 7 | 8 | 9 | 10 |
| --- | --- | --- | --- | --- | --- | --- | --- | --- | --- | --- |
| **Angle(°)** | 115 | 360 | 335 | 256 | 7 | 16 | 15 | 203 | 184 | 51 |
| **Offset Radius（mm）** | 0.075 | 0.065 | 0.023 | 0.100 | 0.024 | 0.015 | 0.011 | 0.009 | 0.021 | 0.046 |
| **No.** | 11 | 12 | 13 | 14 | 15 | 16 | 17 | 18 | 19 | 20 |
| **Angle(°)** | 189 | 112 | 147 | 38 | 224 | 100 | 146 | 300 | 348 | 130 |
| **Offset Radius（mm）** | 0.071 | 0.042 | 0.047 | 0.067 | 0.020 | 0.002 | 0.008 | 0.055 | 0.007 | 0.081 |
| **No.** | 21 | 22 | 23 | 24 | 25 | 26 | 27 | 28 | 29 | 30 |
| **Angle(°)** | 143 | 99 | 98 | 71 | 12 | 27 | 112 | 287 | 9 | 117 |
| **Offset Radius（mm）** | 0.059 | 0.076 | 0.021 | 0.043 | 0.013 | 0.009 | 0.146 | 0.049 | 0.038 | 0.089 |
| **No.** | 31 | 32 | 33 | 34 | 35 | 36 | 37 | 38 | 39 | 40 |
| **Angle(°)** | 35 | 236 | 3 | 81 | 284 | 183 | 184 | 245 | 338 | 294 |
| **Offset Radius（mm）** | 0.013 | 0.033 | 0.127 | 0.054 | 0.055 | 0.074 | 0.045 | 0.049 | 0.006 | 0.034 |
| **No.** | 41 | 42 | 43 | 44 | 45 | 46 | 47 | 48 | 49 | 50 |
| **Angle(°)** | 239 | 284 | 22 | 115 | 358 | 127 | 133 | 265 | 231 | 257 |
| **Offset Radius（mm）** | 0.014 | 0.090 | 0.025 | 0.019 | 0.090 | 0.074 | 0.031 | 0.038 | 0.054 | 0.020 |
| **No.** | 51 | 52 | 53 | 54 | 55 | 56 | 57 | 58 | 59 | 60 |
| **Angle(°)** | 270 | 12 | 113 | 220 | 143 | 238 | 233 | 172 | 92 | 102 |
| **Offset Radius（mm）** | 0.084 | 0.032 | 0.028 | 0.026 | 0.017 | 0.024 | 0.002 | 0.073 | 0.126 | 0.076 |

Subsequently, we used the validated ABAQUS/Explicit models for simulation calculation and extracted the stress-strain curve of the non-uniform spiral material, as shown in **Figure S8(b)**. The results show that the non-uniform structure exhibits smaller platform stress compared to the uniform one, with the reduction in platform stress becoming greater as non-uniformity increases. Specifically, the platform stress decreases by 6.4% and 10.3% for components with standard deviations of 0.5 and 0.25, respectively, when compared with the uniform spiral material.

In terms of structural deformation, the variance in gap sizes leads to a weakened resistance in broader areas, resulting in wrinkling deformation appearing first during the compression process. This phenomenon affects the overall stability of the structure and changes the crushing morphology, as illustrated by the compressed structure at 15% strain in **Figure S8(b)**. Therefore, minimizing non-uniformity induced by processing errors is crucial in the practical fabrication of spiral materials to preserve structural integrity and performance.


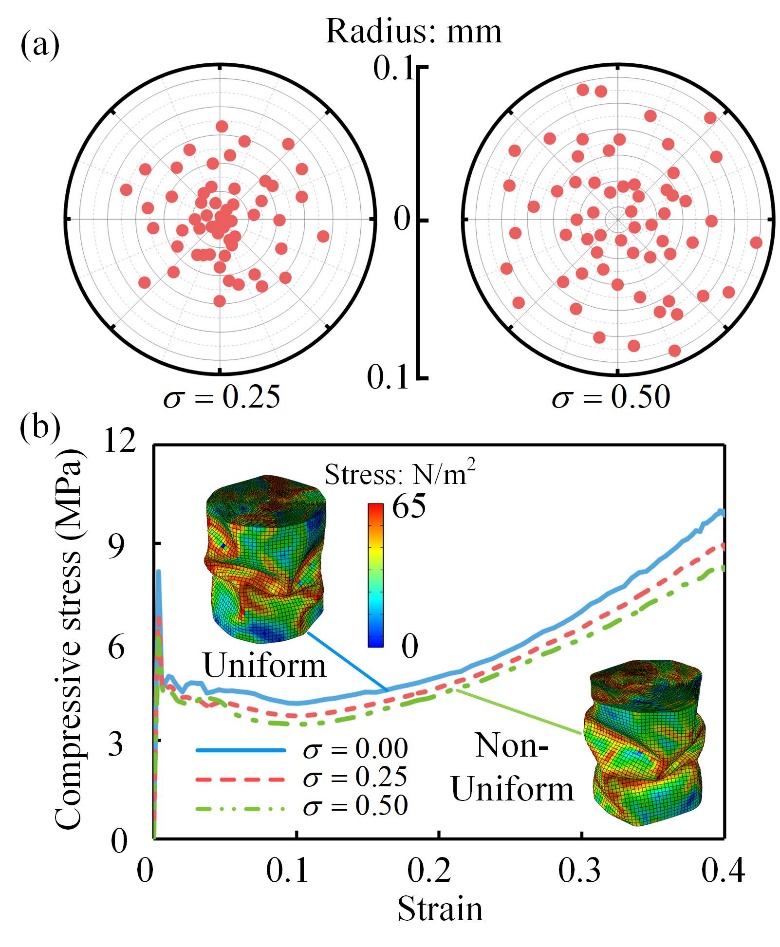


**Figure S8: The effect of non-uniformity in the gap of spiral material on mechanical properties.** (a) The centers of paper rolls in non-uniform roll paper structures with different standard deviations (a=0.25 and a=0.5). (b) Comparison of the stress-strain curves in compression process of spiral materials with different non-uniformity.

**Part 9:** **Acoustic finite element details**

The acoustic performance of the dual-scale spiral materials is evaluated through numerical simulations using COMSOL Multiphysics software, as depicted in **Figure S9**. A three-dimensional (3D) finite element (FE) model is developed for both KQM and PKQM, and 3D numerical calculations are conducted. To enhance computational efficiency, the solid components of KQM and PKQM are simplified as acoustic rigidities due to the significant impedance mismatch between the CEHR wall and air. Consequently, only the FE model of the air domain needs to be established. In the FE model illustrated in **Figure S9**, the blue section represents the pressure acoustics field, where the upper boundary is set as a plane wave incident condition to simulate vertically incident sound waves onto the spiral material from the semi-infinite air domain, with a sound pressure amplitude of 0.01 Pa. The red section corresponds to the thermoviscous acoustics field, where the energy loss caused by air viscosity within the CEHR is calculated. The boundaries connected to the inner walls of CEHR are considered as default acoustic hard boundaries. To capture the continuity between the pressure acoustics field and the thermoviscous acoustics field, the interface between the two sound fields is designated as a pressure-thermoviscous interaction boundary. For meshing, the spiral slit channel and perforated area are divided into free tetrahedral meshes. Through frequency domain research, the distributions of particle vibration velocity, sound pressure, and energy loss density inside and on KQM and PKQM can be obtained.


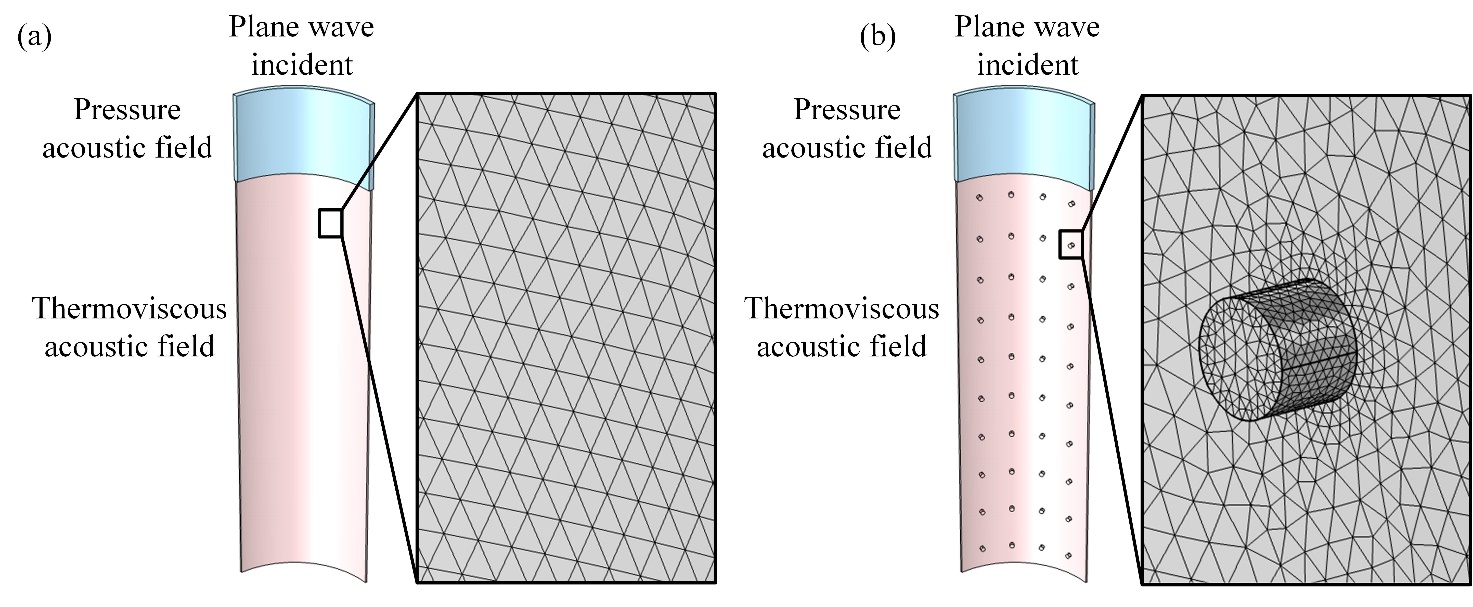


**Figure S9:** **The finite element computational model and mes**h: (a) KQM and (b) PKQM. Free tetrahedral meshes are employed for all domains due to irregularity. The grid resolution at the locations of microperforations has been refined to ensure a minimum grid size that is one-tenth of the structure dimensions.

**Part 10:** **Summary of the sound absorption materials**

**Table S3** Summary and comparation of spiral material with other porous materials

|  | **Spiral materials**  **(in this work)** | | | | | **Aerogel** ([1](#_ENREF_1)) | **Porous ceramic material** ([2](#_ENREF_2)) | **Foamed aluminum** ([3](#_ENREF_3)) | **Natural fiber**  **composite** ([4](#_ENREF_4), [5](#_ENREF_5)) |
| --- | --- | --- | --- | --- | --- | --- | --- | --- | --- |
|  | Xuan paper | Printing paper | | | Packing paper |  |  |  |  |
| **Density**  **[g/cm^3^]** | 0.20 | 0.47 | | | 0.53 | ≈0.002 | ≈0.5 | ≈0.8 | ≈0.13 |
| **Sound absorption (NRC)** | 0.67 | | 0.63 | 0.55 | | 0.76 | 0.48 | 0.12 | 0.20 |
| **Cost** | Low | | | | | High | High | High | Low |
| **Specific strength** | 2 | | 4.2 | 9.9 | | 0.04 | 2.7 | 9.8 | 0.48 |
| **Environmental sustainability** | High | | | | | Low | Low | Low | High |

**References**

1. C. Jia, L. Li, Y. Liu, B. Fang, H. Ding, J. Song, Y. Liu, K. Xiang, S. Lin, Z. Li, W. Si, B. Li, X. Sheng, D. Wang, X. Wei, H. Wu, Highly compressible and anisotropic lamellar ceramic sponges with superior thermal insulation and acoustic absorption performances. *Nature Communications* **11**, 3732 (2020).

2. C. Duan, G. Cui, X. Xu, P. Liu, Sound absorption characteristics of a high-temperature sintering porous ceramic material. *Applied Acoustics* **73**, 865-871 (2012).

3. Y. Kang, J. Zhang, Uniaxial compressive behavior of open-cell and closed-cell aluminum foams. *Materials Review* **19**, 122-124 (2005).

4. W. Yang, Y. Li, Sound absorption performance of natural fibers and their composites. *Science China-Technological Sciences* **55**, 2278-2283 (2012).

5. E. Taban, A. Tajpoor, M. Faridan, S. E. Samaei, M. H. Beheshti, Acoustic absorption characterization and prediction of natural coir fibers. *Acoustics Australia* **47**, 67-77 (2019).

1. * xinchern@126.com (XC); moxiaoli@snu.ac.kr (MXL) [↑](#footnote-ref-1)
